# Non-consciously processed physical activity for survival versus consciously deliberated exercise for health

**DOI:** 10.3389/fpsyg.2023.1181671

**Published:** 2023-06-13

**Authors:** Seppo E. Iso-Ahola

**Affiliations:** Department of Kinesiology, School of Public Health, University of Maryland, College Park, MD, United States

**Keywords:** exercise, physical activity, conscious-non-conscious, automaticity, survival, evolution

## Abstract

Humans evolved to become dependent on physical activity for their survival, but they have not evolved to exercise today. Because survival in modern society is heavily reliant on conscious thinking, most people (54%) have evolved away from physical activity and become occasional exercisers. This transition from non-conscious to conscious processing prevents people from capitalizing on evolution’s wisdom for survival and wellbeing as they consciously deliberate on the utility of health practices to achieve certain outcomes (e.g., weight loss). Unlike in early times, people today have a choice of not engaging in physical activity and yet surviving. As a result, they struggle with the question whether the gains from exercising are greater than losses from not doing it, weighing positive gains and losses against negative gains and losses. Such conscious deliberations, however, can easily be overridden by solving cognitive dissonance (e.g., “exercise is good for my health” vs. “I don’t exercise”) through conscious rationalizations and non-conscious dismissal. Today’s exercise problem can only be solved by individually acquiring the mindset of early times of evolution when the initiation of physical activity was largely a matter of non-conscious thoughts and feelings.

## Introduction

Pursuit of health has caused a major dissonance in modern society. Everyone wants to be healthy, but few are willing to make right lifestyle choices to support their health, leading to the global pandemic of physical inactivity (Kohl et al., 2012). As a result, of the major causes for years of potential life lost before age 65, lifestyle is estimated to account for 53%, followed by environment (21.8%), human biology (16.4%), and the health care system (9.8%) (Powell, 1988). It is further estimated that over 80% of human health is determined by lifestyle, more specifically by four critical activity choices: regular exercise, good nutrition, non-smoking, and moderate alcohol use (Herskind et al., 1996; Iso-Ahola, 2018). On average, the combined effect of these four behaviors is an extension of the lifespan by 7 years compared to only two additional years of life gained if all types of cancers were eliminated from the face of the earth (Ornstein and Erlich, 1989). Of the four, exercise is “the single most important thing” people can do to maintain and improve their health (e.g., Bassuk et al., 2013).

Exercise is an interesting but difficult scientific problem at the individual level. The dilemma mostly stems from cognitive demands exercise poses for individuals. Everyone wants to be healthy without having to work for it, and their conscious thinking readily accommodates this propensity through excuses (Iso-Ahola, 2013). Thus, Festinger’s (1957) cognitive dissonance problem of two conflicting cognitions is a reality for many would-be exercisers: “Exercise is good for me” vs. “I don’t exercise regularly.” An obvious *behavioral* solution to the dissonance would be to start exercising, but since most people (78%) (Blair, 1993) have not been able to do it, they have solved the problem *cognitively* by consciously rationalizing away the importance of exercise and its conflict with other goals. Therefore, it is not surprising that goal conflicts undermine physical activity behavior (Carraro and Gaudreau, 2015).

Dissonance can also be dismissed nonconsciously without any cognitive awareness. After seeing a neighborhood runner countless times, such a reminder about exercising no longer enters conscious awareness and is therefore dismissed automatically, not triggering dissonance in the first place. As Kihlstrom (2008) aptly observed: “As we go about the ordinary course of living, we do not think very hard about anything, and rely on biases, heuristics and other processes that lead us into judgmental error.” Although people can easily solve dissonance by cognitive means, something still does not seem right as personal health issues and others’ behaviors remind them that, at least occasionally, they should go for a walk. It is proposed and explained below why these nagging feelings have their roots in the early years of human evolution.

## Evolved to be physically active to survive

Pontzer (2019) argued that humans “evolved to exercise” and “must move to survive.” This, however, is fundamentally a wrong conclusion. People have not evolved to exercise but instead, they evolved to be physically active and grew dependent on physical activity for their survival. In the early years, simply put, humans had to run fast to catch prey or not eat; or they had to run fast away from predators or perish. In their influential paper, Bramble and Lieberman (2004) argued that endurance running evolved for predator pursuit in early hominids, helping them run mammals to exhaustion to get protein-rich food. As early humans typically covered 9 to 14 kilometers a day (Pontzer, 2019), hunting and gathering dominated subsistence strategies (Raichlen and Alexander, 2020).

After countless repeats of these behaviors over generations, human physiology adapted and as a result, various parts and functions of the body became increasingly more suitable for survival (e.g., more red blood cells, more fatigue-resistant slow-twitch muscle fibers, and faster metabolism) (Pontzer, 2019). In particular, the brain grew bigger in evolutionary time, reorganizing its non-conscious operations to maximize the survival of the species. Physical activity enabled “the massive expansion” of the brain and the brain in turn evolved to reward prolonged physical activity (Raichlen and Alexander, 2017). Physical activity improved neurogenesis (producing new neurons) and neuroplasticity (modifying existing neural pathways), showing why the brain needed physical activity in early times and would still need exercise today (Raichlen and Alexander, 2020).

Based upon her long line of research, Feldman Barrett (2020) similarly concluded that the human brain evolved to regulate physical resources and invest energy wisely to ensure survival: “Your brain’s most important job is to control your body by predicting energy needs before they arise so you can efficiently make worthwhile movements and survive…by prediction, your brain has efficiently prepared you to act” (p. 10, 75). In early times, this brain functioning had clear survival advantages because next actions were initiated non-consciously before cognitive awareness based on past experiences and present situational demands. This is not to say that early humans did not use conscious thinking (e.g., spatial navigation) (Raichlen and Alexander, 2020), but nevertheless the close and reciprocal relationship between non-conscious brain activity and physical activity was essential for survival. However, there was no purposeful physical training for survival. People did not *exercise* daily to become stronger in order to survive.

As conscious-non-conscious processing is central to understanding physical activity and exercise, it is important to clarify these constructs and associated processes. In conscious processing, attended information enters cognitive awareness and is reportable to others; it can be pondered and reoriented (Dehaene, 2014). By contrast, non-conscious processing refers to mental operations regarding feelings and thoughts of which a person is cognitively unaware. These operations are fast, automatic, associative and effortless (Kahneman, 2011). Such “reflective reactions” or “behavioral impulses” (Bargh and Morsella, 2008; Bargh, 2014) affect psychological processes from perception and motivation to behavior. When behaviors become routine and automatic, they are performed with little or no cognitive awareness and grow increasingly habitual. Emerging habits are “chunks” of neural activity located and imprinted in the specific regions and networks of the neocortex, the infralimbic cortex in particular (Graybiel and Smith, 2014).

**FIGURE 1:**
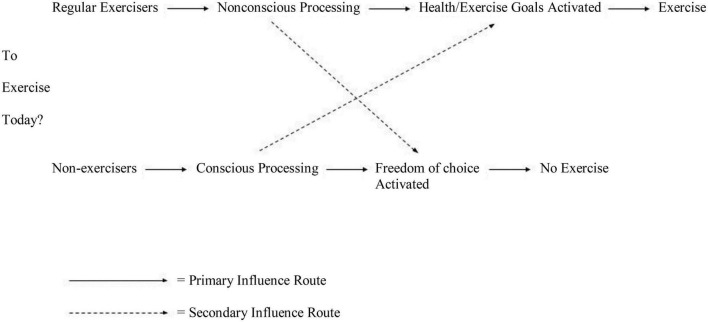
Conscious–non-conscious processing and exercise.

For illustrative purposes, Figure 1 highlights the difference between conscious and non-conscious processing relative to whether or not to exercise on a given day. For regular exercisers, this behavior is prompted by situational cues (e.g., running shoes) that non-consciously and without their awareness activate goals and associated motivation for the usual physical activity (see Custers and Aarts, 2010). For non-exercisers or occasional exercisers, the idea of exercising brings about conscious awareness along with a choice whether to do it or not, which in most cases leads to not engaging in the behavior; occasionally, though, activation of health goals and concerns make them take up physical activity (the secondary route in Figure 1). In a similar vein, regular exercisers occasionally slip because of their activated freedom of choice but most of the time, their goal pursuit is initiated and maintained without conscious awareness.

## Evolved not to exercise

It is important to make a distinction between physical activity for survival in early years and exercise for health today. People certainly have the physiological basis and requirement for exercising for health today (Bassuk et al., 2013), but this foundation lies in the brain’s non-conscious operations underlying physical activity. The issue is that the non-conscious mind no longer has to prepare us to catch prays and run away from predators. Instead, survival today requires conscious thinking, not physical activity.

In many occupations (e.g., white-collar work), survival is a matter of *sitting* in front of a computer and consciously engaging in one’s work. To be sure, there are occupations where relatively little conscious processing is required, but in general, humans have evolved from the non-conscious need for physical activity to survive to the non-conscious (and sometimes conscious) tendency to follow the path of least resistance. As the brain is a “predictive organ” that predicts and prepares action using past experiences (Feldman Barrett, 2020), it has no reason to anticipate considerable energy demands in case of 78% of the population who are sedentary individuals. As Feldman Barrett concluded: “Your actions today become your brain’s predictions for tomorrow, and those predictions automatically drive your future actions” (p. 82).

If one nonetheless chooses to exercise, it becomes a consciously driven activity typically done for extrinsic (e.g., weight loss) reasons, without any immediate survival value. In early times, physical activity had immediate consequences, whereas a lack of exercise today has no near-term ramifications. One can do his/her life’s work for 30–40 years without ever exercising. Long-term negative effects accruing from a lack of regular exercise typically surface later in life, such as non-exercisers having a shorter lifespan by 5 years on average (Blair et al., 1995; Farahmand et al., 2009).

Thus, for most people, the initiation and maintenance of exercise has become a conscious decision to do or not to do it, and this is a key reason why 54% of the population remains “occasional” exercisers and 24% non-exercisers (Iso-Ahola, 2013, 2018). Over time, the non-conscious mind has relinquished its control of exercise over to a conscious decision to engage or not to engage, and when having that choice, most people intentionally opt for not exercising (Iso-Ahola, 2017, 2018), again, because physical activity has no immediate survival value. It is ironic that when conscious decision not to engage is repeated many times, it, too, turns into an automatic default system that is increasingly more difficult to override by occasional conscious attempts to exercise.

Furthermore, exercise has today become a choice among many mundane chores and leisure activities, but it consistently loses the battle of activity choices, as seen in the large percentage of non-exercisers and the fact that this percentage has not changed over many decades (Talbot et al., 2003). Conscious thinking is also good at finding excuses for not exercising. In survey after survey, “lack of time” is the number one reason for not exercising while, at the same time, people find nearly 5 h a day to watch TV and 5 h to peer at their smart phones (Grontved and Hu, 2011; Atus, 2018; Edwards, 2018). In early times, the non-conscious mind handled physical activity without giving people any choice about it, but now, with the conscious mind in charge, people have an option of not exercising and yet surviving. Thus, it is easy for people to ask: Why should I subject myself to daily cognitive and physical strain since I can survive without having to endure such hardship?

In modern society, there is neither physical-activity-based nor exercise-based survival, only conscious-mind-based survival. One consequence is that people are increasingly developing into dualists who separate mind and body, viewing the body as a “vessel” or tool for the mind’s interactions with the world. Forstmann et al. (2012) showed experimentally that dualistic beliefs not only had negative effects on health attitudes and actual health behaviors but importantly, exposures to health-constraining behaviors (i.e., images of unhealthy activities and individuals) increased dualistic beliefs. Common and unavoidable images of sedentary and overweight individuals in today’s society, for one thing, are likely to foster dualistic beliefs and therefore adversely impact health behaviors like exercising. Dualistic beliefs are aligned with conscious-mind-based survival today.

It has been argued that to get demanding health behaviors (e.g., exercise) consistently accomplished, they must be delegated to non-conscious processing because conscious deliberations of pros and cons often make people choose the path of least resistance (i.e., cons win) (Iso-Ahola, 2022). However, if exercise has been repeated to the point where no conscious thinking is required for its initiation and maintenance, it will get done most of the time as the brain predicts and prepares today’s exercise using yesterday’s physical activity demands. The more frequently an activity is repeated, the easier it becomes to perform. This, then, is the irony and paradox of the law of least effort: people initially resist demanding health behaviors, but once they have been repeated many times, they become easier to undertake, and with continuous repeating and time, they turn into non-demanding everyday behaviors. A problem, of course, stems from mental strength required to repeat the behavior long enough so that it becomes entrenched in automaticity before giving up. This is an important area for future research.

However, social environments work against it, as cues for exercise and physical activity are almost non-existent, but cues for sedentary activity abound (Iso-Ahola, 2018). Furthermore, both experimental and non-experimental evidence has shown that such cues as fast-food logos become cues for temptations and non-consciously activate time saving and impatience, thereby reinforcing the idea of not having time for exercise (Zhong and DeVoe, 2010; DeVoe et al., 2013; Iso-Ahola and Miller, 2016). Simultaneously, cues for non-demanding unhealthy behaviors are abundant in social environments, thus overpowering infrequent cues for demanding health behaviors and choices. It is then not surprising that avoiding sedentary behaviors requires more cortical resources than avoiding physical activity (Cheval et al., 2018b) and that it is easier to non-consciously motivate people not to be active than motivate them to become active (Iso-Ahola and Miller, 2016). In brief, demanding health behaviors face a formidable, two-pronged opposition: individual propensity to follow the path of least resistance on the one hand and activation of abundant cues for unhealthy behaviors in social environment on the other.

It is important to note, however, that a significant segment of the U. S. population (22%) exercises regularly. They have been able to turn an initially conscious behavior into a predominantly non-consciously driven activity. They do it day after day prompted by situational cues, thereby having delegated self-control of the behavior to contextual reward cues in routine daily environments (Bargh, 2017). Automatic repeating of physical activity enabled by non-conscious processing eliminates a sense of obligation associated with behavioral engagement (i.e., no chance to *think* and weigh the choice of doing or not doing an activity). Automaticity also makes this originally demanding activity into a less and less demanding behavior in the long run (Iso-Ahola, 2022).

Furthermore, when an activity has been designated as the one that is performed no matter the circumstances (Houser-Marko and Sheldon, 2006), effort expenditure for activity execution becomes its own reward (Wang et al., 2017; Cheval et al., 2018a), thus eliminating mental effort as inherently aversive and costly (Shenhav et al., 2017). Even if exercisers originally made a negative or forced choice to engage in a physical activity, the activity turns into an intrinsically motivated behavior with repeats and time (i.e., no need to *think* because they now love it) (Iso-Ahola, 2013). They have also learned to use immediate intrinsic rewards (i.e., enjoyment) as proximal subgoals to stay on the track of maintaining their long-term goal pursuit in exercise activity (Woolley and Fishbach, 2017). Intrinsic motivation promotes non-conscious processing and thereby the habitualness of physical activity (Iso-Ahola and St. Clair, 2000; Gardner and Lally, 2013).

It has been suggested that physical environments can promote physical activity through enticing outdoor settings. However, such a positive effect is small as the number of regular exercisers increases only by 10 to 32% from the national average of 22% in Colorado, arguably the most alluring environment for physical activity. If the increase is so small in the most attractive environment, it is hardly surprising that an equivalent increase is non-existent in less alluring environments (Iso-Ahola, 2018), thereby further attesting to the importance of the human mind as an ultimate decider of physical activity participation.

## A new conceptualization of gains and losses

Given that the wide and deep effects of exercise on health and mortality have been well communicated to the public, it is surprising that only a small section of the U. S. population exercises regularly. As Bassuk et al. (2013) and others (Blair, 1993; Lee et al., 2012) have shown, regular exercise significantly reduces the prevalence of heart disease, stroke, diabetes, and most forms of cancer; improves endocrine, immune, and musculoskeletal systems; extends the lifespan by 5 years on average (Lee et al., 1995; Farahmand et al., 2009). It also enhances cognitive functioning through enhanced neuroplasticity and neurogenesis (Bezzola et al., 2011; Erickson et al., 2011). Neurogenesis in turn keeps the brain fit for learning, thus underscoring the “use-it-or-lose-it” principle (Shors et al., 2012, Shors, 2014). There is not one part of the human body that would not benefit from exercise. Indeed, “exercise is medicine,” as the national motto declared a few years ago. It is obvious that exercise greatly improves the human condition through improved health and extended lifespan.

**FIGURE 2:**
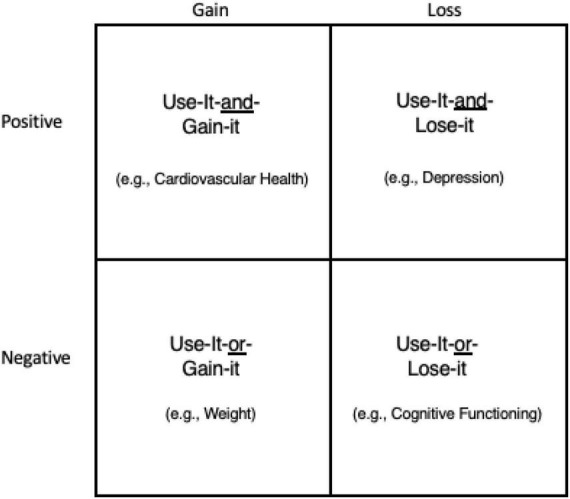
Positive and negative gains and losses from exercising for health.

Despite these well-documented benefits of exercise, 54% of the population (“occasional” exercisers) struggle consciously with engagement in this health behavior, asking: Are the gains from exercising greater than the losses from not doing it? As a result, they find themselves in one of the following psychological conditions or any combination of them (Figure 2):

(A) Use-it-and-gain-it (positive gains).
(B) Use-it-and-lose-it (positive losses).
(C) Use-it-or-gain-it (negative gains).
(D) Use-it-or-lose-it (negative losses).

In the first two cases, both gains (A, e.g., improved cardiovascular condition) and losses (B, e.g., reduced depression) from using it are positive, whereas in the latter two cases, both gains (C, e.g., increased weight), and losses (D, e.g., decreased bone density) from not using it are negative. If would-be exercisers saw the sum of A and B greater than that of C and D for maintaining and improving health, they might be persuaded to start an exercise program. They would expect to be positively reinforced by the positive gains and losses, whereas those motivated by C and D would be negatively reinforced by avoiding negative gains and losses.

A major problem, of course, is that in both cases, gains achieved and losses avoided typically take many months or years to be realized, which will surely disappoint individuals motivated to achieve these outcomes. Hence, it is not surprising that research has shown that of those who start an exercise program today, only about 20% will continue it 5 weeks later (Armitage, 2005). An additional problem for those motivated by C and D is that many of them would be exercising under threat, fear, or risk perception: “do it or else.” Evidence, however, does not support the effectiveness of these types of negative motives for changing health behaviors (Schwarzer, 2001; Sniehotta et al., 2005), but more research needs to be done on fear-related barriers to physical activity, especially among adults with overweight and obesity (Hamer et al., 2021).

The new conceptualization presented in Figure 2 demonstrates the cognitive difficulty people face when they try to consciously decide whether to start and maintain health practices in general and exercise behavior in particular. It is no wonder why most people remain “occasional” exercisers as they consciously weigh potential gains and losses. Furthermore, in each of the four scenarios, exercise is linked to a product or a long-term outcome, that is, extrinsic contingencies (e.g., rewards, sanctions).

In general, it is well established that health-related goal pursuits can be sustained in the long term only if they are non-consciously driven (Marteau et al., 2012; Bargh, 2017), having been designated as activities to be done no matter what (Houser-Marko and Sheldon, 2006) and reinforced by intrinsic rewards (i.e., an activity done for its sake) (Deci and Ryan, 2000). Extrinsic contingencies and rewards, in contrast, are indicative of exercise being a means toward an end (e.g., weight loss) rather than an end itself. If exercise were an end itself, it would be a lifestyle, not an activity or a program to achieve something. When health practices are undertaken as a means toward an end, they become outcome-oriented and include a choice of not doing them. This is a major reason why conscious deliberations will not be able to turn most people into regular exercisers (Iso-Ahola, 2013, 2018).

It remains to be determined by future research how the four conditions delineated in Figure 2, individually or in combination, will help persuade people to initiate and maintain their exercise activity. As thinking of gains and losses requires conscious awareness and focus, it may be asked: Would some of the cells of Figure 2 be more conducive to continuous repeating of an exercise activity later and thus make it driven by non-conscious processing? How does the transition from conscious thinking of gains and losses to non-conscious processing of activity maintenance occur in real life? In this regard, it would also be important to investigate group differences (e.g., gender and age), as well as societal and cultural differences. For example, gains and losses may have varying meanings for older and younger individuals.

## Strategies for change

An irony of evolution is that thinking as its byproduct has enabled humans to produce breath-taking technological discoveries to make life easier, but not healthier and longer. Since conscious thinking alone cannot solve the problem of long-term maintenance of health practices for most people, evolution’s main tool for human survival, the brain and its non-conscious operations, has to be employed to solve the problem. However, non-conscious processing cannot solve the problem alone either– until activity involvement becomes automatically initiated and maintained non-consciously by situational cues. Long-term maintenance of physical activity necessitates that self-regulation of behavior is relegated to contextual reward cues (Bargh, 2017). However, a problem is that non-conscious processing is vulnerable to the influence of those situational cues that are aligned with passive activities (e.g., a remote control for TV watching) (Kubey and Csikszentmihalyi, 2002; Iso-Ahola, 2018).

The basic principle of non-conscious processing and behavior is the cue-behavior link (Kihlstrom, 1987; Bargh, 2006, 2017, 2021). That is, when an exercise activity is consistently prompted by a situational cue, this cue-behavior link becomes stronger and more automatic with every enactment of the behavior. However, is it strong enough to override the other cue-behavior links that are also strong and rewarding but geared toward passive activities? This is a fruitful area for future research.

All the above suggests that a key is the interplay of conscious and non-conscious processing (Baumeister and Bargh, 2014). The former can serve the latter through conscious arrangements of one’s environment in two principal ways: (1) Many and easily noticed situational cues for exercise are consciously made available (e.g., sneakers put out, visibly ready for a morning run/walk). These cues are critical for non-conscious processing and strengthening the cue-behavior link (i.e., maintenance of physical activity in the long term). (2) At the same time, competing cues for passive activities are removed, for example, by placing televisions in the house where they cannot immediately be seen or readily accessed.

These intentional manipulations of situational cues can help make an exercise activity a default system (Iso-Ahola, 2018), not unlike financial default systems (e.g., a certain percentage of one’s monthly salary automatically deducted for pension) recommended and “nudged” by behavioral economists (Thaler and Sunstein, 2008). Although it is probably easier to opt out of an exercise default system than the financial one, people do not generally opt out of strong default systems (Thaler and Sunstein, 2008; Bargh, 2017; Chapman, 2019). Ironically, actively opting out is hard work and cognitively straining, and therefore avoided. However, as there is a paucity of research on the development and maintenance of exercise default systems, empirical testing of these ideas is needed (Hagger, 2019).

A second major strategy is to use “implementation intentions” (Gollwitzer, 1999; Gollwitzer and Sheeran, 2006). These are specific if-then plans of “when,” “where,” and “how” to engage in an activity. It has been suggested that a fourth component, “with whom,” be added to the if-then plans when building an “exercise infrastructure” (Iso-Ahola, 2017). Evidence indicates that people are more likely to maintain their exercise activity if they can do it with friends or spouses, especially in early stages of exercise programs (Anderson et al., 2006; Berli et al., 2018). Similarly, social connections, support, and networks correlate with physical health and well-being (Holt-Lunstad, 2021; Kok et al., 2013; Rook, 2015). An important advantage of implementation intentions, as research has shown, is that they rapidly become non-conscious (Gollwitzer et al., 2011).

Even though the implementation intentions are consciously formed in the beginning stages of activity programs, they quickly become embedded into situational cues so that one does not have to think when, where, how, and with whom to exercise. These specific if-then plans are already included in the cue that prompts the behavior. As a whole, then, it is evident that non-conscious processing is the one that gets the job done. Without it, people cannot become regular exercisers. Of course, conscious thinking is still around and can veto a morning run/walk if there are compelling reasons for aborting it on a given day (Baumeister et al., 2011; Iso-Ahola, 2013). But most of the time, successful execution of a behavior is reliant on non-conscious processing with no or minimal input from conscious processing (Bargh, 2021).

One straightforward solution would be for people to make a permanent, long-term decision to exercise no matter what. Such a decision would remove any conscious choice about the behavior, which would expedite the transition from conscious to non-conscious processing. This decision, however, is difficult for most people and as a result, only 22% of the U. S. population are regular exercisers. But there is a group of individuals called “self-as-doers,” who make such a decision not just in relation to exercise but all of their behaviors. Their main concern is with enactment of the behavior, not with outcomes, rewards, or even enjoyment; their focus is simply getting the task or activity done. Houser-Marko and Sheldon (2006), who introduced the concept, showed that the mindset of doing a behavior no matter what correlates positively with persistence, goal attainment and maintenance of long-term behaviors like exercise.

Importantly, the self-as-doer mindset removes a choice about the behavior and minimizes opportunities for conscious deliberations regarding the value and utility of doing the behavior (Iso-Ahola, 2013, 2017). It therefore puts non-conscious processing in charge of getting the behavior done. In the absence of an unwavering commitment or decision to carry out an exercise activity no matter what, individuals can manipulate situational cues in their environment and employ implementation intentions to facilitate non-conscious processing and more automatic execution of exercise behavior. Ironically, this takes people back to early times and helps them capitalize on evolution’s wisdom for human survival and wellbeing.

## Conclusion

Although exercise is the single most important thing people can do for their health, the majority has nevertheless degenerated and devolved into non-exercisers, or “occasional” exercisers at best. It is important to make a distinction between physical activity for survival in early times and exercise for health today. This evolved drifting away from physical activity that underpinned survival in early times has shifted the focus on conscious deliberations regarding exercising in modern society. As a result, most people find themselves weighing benefits of exercising against losses from not doing it (positive gains and positive losses vs. negative gains and negative losses), causing them to struggle with cognitive commitment to regular exercise. Solving the individual exercise problem, therefore, necessitates the interplay of conscious and non-conscious processing so that the conscious mind is employed to facilitate the non-conscious maintenance of involvement in physical activity. This mindset, ironically, takes people back to evolution’s wisdom for individual survival and wellbeing in early times.

## Author contributions

The author confirms being the sole contributor of this work and has approved it for publication.
